# A56 TUFT CELL RESPONSES DURING ACUTE- AND LATE-STAGE *GIARDIA* INFECTION

**DOI:** 10.1093/jcag/gwab049.055

**Published:** 2022-02-21

**Authors:** O Sosnowski, T Allain, E Fekete, D M Mckay, A Buret

**Affiliations:** 1 Biological Sciences, University of Calgary, Calgary, AB, Canada; 2 Physiology & Pharmacology, Uni. Calgary, Calgary, AB, Canada

## Abstract

**Background:**

Epithelial tuft cells can detect and respond to enteric infections and appear to help clear some intestinal parasites. Through tuft cell luminal surface receptors, tuft cells can sense ligands directly supplied by a parasite, or indirectly *via* excretory/secretory products. We hypothesize that microbiome alterations may also modulate tuft cell-derived gut responses. Tuft cells release the alarmin cytokine IL-25 which, upon acting on type 2 innate lymphoid cells (ILC2), ultimately lead to tuft and goblet cell hyperplasia. Upon helminth infections, tuft and goblet cell hyperplasia occurred concurrently, and coincided with the peak of infection. The role of tuft cells in infections with the enteric protozoan parasite *Giardia sp.* is unknown. The aim of our study is to characterize how tuft cells may be implicated in the pathophysiology of giardiasis, in an attempt to uncover novel regulatory pathways of intestinal physiology.

**Aims:**

In this study, we aim to characterise the tuft cell response to *Giardia* infection during acute and late stages of infection and to assess goblet cell hyperplasia.

**Methods:**

5–7-week-old C57BL/6 mice and tuft cell-deficient mice ( *Pou2f3*^*-/*-^) were orally gavaged with 5x10^4^*Giardia muris* trophozoites and scarified at days 4, 11 and 21 post-infection. Parasite burden was assessed in the duodenum. Immunofluorescence (IF) staining of doublecortin-like kinase 1 (DCLK1) – a marker of tuft cells – was performed on C57BL/6 mice jejunum tissue sections and the number of tuft cells was quantified. Goblet cells were quantified in PAS/AB-stained jejunum sections. Quantitative PCR (qPCR) was performed on *Dclk1*, the epithelial secretory cell transcription factor *Atoh1,* and the mucus gene *Muc2*.

**Results:**

*G. muris* infected C57BL/6 mice displayed high parasite load at days 4 ( *p*<0.05) and 11 ( *p*<0.05), with no or low parasite burden at day 21 ( *p*<0.05). *Pou2f3*^*-/*-^ mice showed less robust parasite burden at days 4 and 11, and similar low parasite burden at day 21, compared to WT mice. At day 21, tuft cell (DCLK1+) counts ( *p*<0.05) and *Dclk1* mRNA expression levels were increased in *Giardia* infected mice in the jejunum. Goblet cell number and *Atoh1* and *Muc2* expression were increased at day 4 post-infection.

**Conclusions:**

The data demonstrate that tuft cells expand late in *Giardia* infection, suggesting that upon parasite infection, tuft cells may possess roles in tissue repair or clearance of infection. Tuft cell-deficient mice ( *Pou2f3*^*-/*-^) had lower parasite burden early in *Giardia* infection, counter-intuitively suggesting that tuft cells may facilitate trophozoite colonization, further highlighting the novelty of these findings. The crosstalk between tuft cells, *Giardia*, and other host responses during acute and late stages of infection remain to be fully characterised.

**Funding Agencies:**

NSERC

